# Cinétique de la troponine Ic et valeurs seuils pour le diagnostic d'infarctus du myocarde après chirurgie cardiaque sous circulation extracorporelle

**Published:** 2012-12-29

**Authors:** Samy Kallel, Anwar Jarraya, Maged Ellouze, Imed Frikha, Abbdelhamid Karoui

**Affiliations:** 1 Service d'anesthésie et de réanimation, CHU Habib Bourguiba, Tunisie; 2 Service de chirurgie thoracique et cardiovasculaire, CHU Habib Bourguiba, Tunisie

**Keywords:** Cinétique, troponine Ic, cutoff, chirurgie cardiaque, infarctus du myocarde, Kinetic, cTnI Ic, cutoff, cardiac surgery, myocardial infarction

## Abstract

**Introduction:**

L'objectif de ce travail était d’étudier la cinétique de la Troponine Ic (TnIc) après chirurgie cardiaque sous circulation extracorporelle (CEC) et établir des valeurs seuils de TnIc pour le diagnostic d'infarctus du myocarde (IDM) post opératoire. Il s'agissait d'une étude prospective type cohorte observationnelle.

**Méthodes:**

Nous avons inclus 37 patients âgés de plus de 18 ans proposés pour chirurgie valvulaire ou pontage aorto coronarien sous CEC. Nous avons suivi la cinétique de TnIc par des dosages immunoenzymatique sur mini-vidas^®^ avant et après la CEC, à H4 et H12 postopératoire puis tous les jours les 4 premiers jours. Le cutoff pour le diagnostic d'IDM post opératoire a été défini comme la valeur moyenne de troponine + deux déviations standards des patients indemnes de complications cardiaques.

**Résultats:**

Les valeurs de TnIc en préopératoire étaient toutes inférieures au seuil de détection de la méthode de dosage (<0,01µg/l). Les valeurs de TnIc augmentent en postopératoire immédiat pour atteindre un maximum à H4 puis diminuent progressivement pour se normaliser après 4 à 5 jours. Les valeurs seuils ont été déterminées pour H0, H4,H12, H24, H48, H72, H96 et ont été respectivement 1.36, 2.58, 3.1, 3.23, 2.13, 1.53, 1.17 pour la chirurgie coronaire et 3.75, 5.39, 4.22, 3.41, 1.65, 1.3 1.19 pour la chirurgie valvulaire.

**Conclusion:**

La connaissance de la cinétique de TnIc lors de chirurgie cardiaque non compliquée et la fixation de valeur seuil ou Cutoff permet aux cliniciens de distinguer entre dommage myocardique secondaire à la chirurgie et IDM.

## Introduction

Après une chirurgie cardiaque avec circulation extracorporelle (CEC) les critères habituels de diagnostic d'infarctus du myocarde (IDM) postopératoire s'avèrent peu sensibles et peu spécifiques [1]. Actuellement, une protéine cardiospécifique: la troponine Ic, est considérée comme marqueur de choix pour porter le diagnostic d'infarctus myocardique péri opératoire [2]. Or dans ce type de chirurgie, l’élévation du taux des troponines est quasi constante chez tous les patients même ceux qui n'ont pas présenté d'IDM. Cette élévation de la TnIc est expliquée par les manipulations directes du muscle cardiaque ou par une protection myocardique insuffisante. Ceci rend le diagnostic d'IDM après une chirurgie cardiaque de plus en plus difficile et incite à établir des “cutoff” ou des valeurs seuils au-delà desquels le diagnostic d'IDM post opératoire peut être retenu.

Le cutoff peut être défini comme la valeur moyenne plus deux déviations standards des patients indemnes de complications cardiaques ou comme la valeur ayant la meilleure sensibilité et spécificité en réalisant la courbe ROC. Ces valeurs seuils ou cutoff sont spécifique à chaque équipe et à chaque laboratoire car le calibrant utilisé par les différentes trousses de réactifs n'est pas encore standardisé, de sorte que les résultats des techniques varient d'un fabricant à l'autre.

Dans cette étude, nous nous sommes proposés d’étudier la cinétique de la TnIc post CEC non compliquée d'IDM postopératoire et d’établir des valeurs seuils "cutoffs " propre à notre équipe nous permettant de faire le diagnostic de l'IDM post opératoire.

## Méthodes

Nous avons conduit une étude prospective type cohorte, observationnelle, sans modification de la stratégie thérapeutique incluant les patients opérés d'une chirurgie cardiaque programmée ou semi urgente sous circulation extra corporelle (CEC) et qui ont été pris en charge en postopératoire dans l'unité de réanimation postopératoire du Service de chirurgie thoracique et cardiovasculaire du CHU HABIB BOURGUIBA de SFAX durant une période de 7 mois.

Les critères d'inclusion ont été: l’âge supérieur à 18 ans et la chirurgie cardiaque programme ou semi urgente avec clampage aortique et CEC. Nous n'avons pas inclus les patients opérés en urgence et les patients ayant eu un IDM dans la semaine précédant l'intervention ou ayant une insuffisance rénale chronique ainsi que les cas de Chirurgie combinée: pontage aorto-coronarien (PAC) et remplacement valvulaire (RV). Nous avons exclus les cas de reprise chirurgicale pendant les quatre jours suivant l'intervention ainsi que les cas de décès per opératoire ou pendant les quatre jours suivant l'intervention ne permettant pas de suivre la cinétique des marqueurs. Nous avons exclu aussi les patients ayant présenté un IDM post opératoire défini par l'association d'une reélévation après une baisse de la troponine I cardiaque et d'au moins un des critères suivants: symptômes d'ischémie myocardique, apparition d'une nouvelle onde Q (durée > 0,04 sec et amplitude > 30% amplitude du QRS), ou une nouvelle anomalie ECG évocatrice d'ischémie myocardique dans au moins deux dérivations contiguës.

Notre protocole d'anesthésie a été standardisé pour tous les patients La conduite pré anesthésique comportait un jeûne d'au moins six heures avec une prémédication anxiolytique par voie orale à l'Hydroxyzine. Les médicaments reçus par le patient ont été managés selon les recommandations habituelles.

Au bloc opératoire, les patients ont été installés en décubitus dorsal sur un matelas chauffant. Après la mise en place des éléments de surveillance per opératoire, un cathéter a été inséré dans l'artère radiale. L'induction de l'anesthésie a été réalisée par l'association d'un hypnotique (Midazolam (Hypnovel^®^) à la dose de 0,1 mg/kg), d'un analgésique morphinique (Rémifentanyl (Ultiva^®^) à la dose de 1,5 µg/kg sur une minute) et d'un agent curarisant (Cisatracurium (Nimbex^®^) à la dose de 0,2 mg/kg). Après intubation orotrachéale, les patients ont été ventilés en mode contrôlé avec un mélange d'oxygène et d'air à 50%, un volume courant de 8 à 10 ml/kg et une fréquence respiratoire pour maintenir une PaCO2 entre 35 et 40 mm Hg.

L'entretien de l'anesthésie a été assuré par l'association du Midazolam (0,05 mg/kg/h), du Rémifentanyl (0,2 à 0,4 µg/kg/min) et de Cisatracurium (0,1 mg/kg/h). Du Sévoflurane a été utilisé pour le préconditionnement myocardique en cas de PAC. La surveillance per opératoire a été assurée par un électrocardiogramme avec 5 dérivations, une saturation pulsatile en oxygène, un capnogramme, une pression artérielle invasive, une pression veineuse centrale et la diurèse. L'antibioprophylaxie a été à base de 2g de Céfazoline ou de Vancomycine 15 mg/kg sur une heure en cas d'allergie.

La dose d'héparine a été de 300 UI/kg, injectée par l'anesthésiste dans le cathéter central, pour atteindre un temps de coagulation activé (ACT) supérieur à 400 secondes. Après vérification d'une anti coagulation efficace une circulation extracorporelle a été mise en place. La circulation extracorporelle a été standardisée pour tous les patients. Le débit de la pompe a été maintenu à 2,4 l/min/m2 avec comme objectif le maintien d'une pression artérielle moyenne entre 60 et 80 mm de Hg pendant toute la CEC. La température a été maintenue à 36°C. La protection myocardique consistait en une perfusion par voie antérograde de 0,5 l d'une solution de cardioplégie cristalloïde comportant une ampoule de chlorure de potassium à 7,5% et une demi-ampoule de magnésium à 15%. L'utilisation de chocs électriques internes (CEI), en cas de fibrillation ventriculaire en sortie de CEC, a été notée. En fin de CEC, nous avons administré du sulfate de protamine par voie intraveineuse pour atteindre un ACT inférieur à 160 secondes.

En post opératoire, tous les patients ont été transférés à l'unité de réanimation postopératoire où ils ont reçu une sédation au propofol pendant 2 à 4 heures, une analgésie à la morphine (20 à 40mg/24h) et au paracétamol (4gr/24h). Le traitement anticoagulant a été débuté à la 6ème heure en l'absence de saignement et de troubles de l'hémostase. La protection gastrique a été assurée par l'omeprazole. L'extubation trachéale a été envisagée dès que des critères prédéfinis ont été réunis. Les patients ont eu systématiquement des séances de ventilation non invasive (VNI) toutes les six heures. Le saignement postopératoire a été évalué par la mesure du volume total des drainages thoraciques. Durant les 4 premiers jours de la période post opératoire tous les patients ont eu un ECG biquotidien en plus de la surveillance électrocardioscopique continue.

Pour le dosage de la TnIc, tous les prélèvements ont été effectués dans des tubes à l'héparinate de lithium puis envoyé immédiatement au laboratoire où ils ont été centrifugés à 3000 tours par minute pendant 10 minutes. Le dosage de la TnIc a été immédiat sans recours à la conservation. Il a été réalisé par méthode immunoenzymatique “ELFA” sur mini-vidas^®^ de Biomérieux. Le domaine de mesure du coffret s’étend de 0,01 à 30 µg/l, au delà de cette valeur, il est recommandé de pratiquer une dilution avec un sérum dont les taux de TnIc < 0,01. Le seuil décisionnel recommandé par le fournisseur est de 0,11 µg/L. Coefficient de variation a été de 4,1%. Ce dosage a été réalisé avant et après la CEC, à H4 et H12 postopératoire puis tous les jours les 4 premiers jours.

Pour établir la valeur seuil de positivité de la TnIc pour le diagnostic d'infarctus péri opératoire, l'utilisation de la courbe ROC n’était pas possible vue que nous n'avions pas de patients présentant un IDM péri opératoire précoce. Cependant, le “cutoff” dans notre étude a été défini, pour chaque population, comme la valeur moyenne plus deux déviations standards des patients indemnes de complications cardiaques.

L'analyse statistique a été réalisée avec le logiciel SPSS 18.0 pour Windows. Les données sont exprimées en moyenne ± déviation standard (DS). Les variables quantitatives ont été exprimés en moyennes et écart-type “moyenne±écart-type” et ont été comparées par le test de Student et le test non paramétrique U de Mann-Whitney chaque fois que la distribution n'est pas gaussienne ou que la taille de l'un des groupes est faible (<10). Le test ANOVA et la corrélation linéaire de Pearson ont été aussi utilisés. Les variables qualitatives ont été décrites par leur effectif et leur proportion “effectif(%)” et ont été comparées par le test de Chi-2 ou par le test des probabilités exactes de Fisher en fonction des conditions de validité. Les différences entre les résultats ont été considérées significatives pour une valeur de p < 0,05.

## Résultats

Dans cette étude 37 patients ont été inclus. Deux patients ont été exclus suite à leurs décès avant le quatrième jour post opératoire. Les 35 patients restants ont été répartis sur deux groupes: population coronaire (22 patients) et population valvulaire (13 patients).

Les caractéristiques démographiques sont résumées dans le Tableau 1. La population coronaire a été plus âgée (61,5±10 ans contre 42,1±13 ans pour la population valvulaire soit p=0.001). Le sex-ratio a été de 1,2. Nous observons une prédominance masculine dans la population coronaire 63,6% contre 38,5% pour la population valvulaire. Concernant les comorbidités associées (Tableau 2), la population coronaire présentait plus de surcharge pondérale, d'IDM récent et de diabète. Alors que la population valvulaire présentait plus d'ACFA.

**Tableau 1:** Caractéristiques démographiques des patients étudiés

Variables	Population générale	Population coronaire	Population valvulaire	P
Nombre de patients	35	22(63%)	13(37%)	
Age (années)	54,3±15	61,5±10,2	42,1±13,9	0,001
Sexe (homme)	19(54,3%)	14(63,6%)	5(38,5%)	NS
Poids (kg)	64,9±11	67,4±10,6	60,6±10,8	NS

NS : non significative

**Tableau 2:** Comorbidités des patients étudiés

Variables	Population générale	Population coronaire	Population valvulaire	P
IMC > 25	13(37%)	11(50%)	2(15,4%)	*0,041*
HTA	12(34,3%)	10(45,5%)	2(15,4%)	NS
ACFA	3(8,6%)	0	3(23,1%)	*0,044*
Maladie vasculaire périphérique	1(2,9%)	1(4,5%)	0	NS
IDM < 3mois	11(31,4%)	11(50%)	0	*0,002*
Diabète	10(28,6%)	9(40,9%)	1(7,7%)	*0,036*
BPCO	3(8,6%)	2(9,1%)	1(7,7%)	NS
FEVG < 50%	4(11,4%)	3(13,6%)	1(7,7%)	NS
HTAP > 60	3(8,6%)	1(4,5%)	2(15,4%)	NS
IRC	8(22,9%)	7(31,8%)	1(7,7%)	NS
EuroSCORE	4±2,1	3,7±1,9	4,4±2,4	NS
EuroSCORE > 6	8(23%)	3(13,6%)	5(38,5%)	NS

NS : non significative

En per opératoire, la durée de la CEC, la durée de clampage aortique (CA), la durée de la chirurgie et la quantité de cardioplégie ne diffèrent pas de manière significative entre les deux groupes étudiés. Les caractéristiques de la CEC sont résumées dans le Tableau 3.

**Tableau 3:** Caractéristiques de la circulation extracorporelle

Variables	Population générale	Population coronaire	Population valvulaire	P
Durée CEC (min)	93,6±28,9	94,6±28,5	91,8±30,8	NS
Durée CA (min)	63,9±22,8	60,1±18,6	70,8±28,6	NS
Quantité cardioplégie (ml)	2008±458	2060±527	1921±313	NS

NS : non significative

De même, la comparaison des incidents per opératoires (transfusions, fibrillation ventriculaire, CEI, stimulation ou CPIA) selon la procédure chirurgicale ne révèle pas de différence significative. Le Tableau 4 résume les complications per opératoires.

**Tableau 4:** Complications peropératoires

Variables	Population générale	Population coronaire	Population valvulaire	P
Transfusion	14(40%)	10(45,5%)	4(30,8%)	NS
Nombre d'unités transfusées	0,9±1,7	1,2±2	0,5±0,9	NS
Fibrillation ventriculaire	15(42,9%)	9(40,9%)	6(46,2%)	NS
Choc électrique interne	0,6±0,9	0,5±0,6	0,9±1,2	NS
Stimulation	8(22,9%)	4(18,2%)	4(30,8%)	NS
CPIA	0	0	0	NS

NS : non significative

En post opératoire, nous avons pu extuber 94,3% de nos malades avant la sixième heure. La comparaison du délai d'extubation, du délai de dédrainage et des durées d'utilisation des catécholamines entre les deux populations ne révèle pas de différence significative. Les données post opératoires sont résumées dans le Tableau 5.

**Tableau 5:** Données postopératoires

Variables	Population générale	Population coronaire	Population valvulaire	P
Délai de dédrainage	2,4±1,05	2,6±1,29	2,2±0,38	NS
Délai d'extubation (heures)	4±3,63	3,8±2,81	4,2±4,83	NS
Durée catécholamine (jour)	1,4±1,31	1,6±1,37	1±1,15	NS
Durée noradrénaline (jour)	1,3±1,35	1,6±1,37	0.9±1,21	NS
Durée dobutamine (jour)	0,4±0,81	0,4±0,90	0,4±0,65	NS

NS: non significative

Les valeurs de TnIc en préopératoire étaient toutes inférieures au seuil de détection de la méthode de dosage (<0,01µg/l). Les moyennes de TnIc à l'arrêt de la CEC (H0), à H4, H12, H24, H48, H72 et H96 postopératoire sont résumées dans le Tableau 6.

**Tableau 6:** Les moyennes des TnIc en µg/L aux différents temps étudiés pour la population générale, la population coronaire et la population valvulaire

Heure	HO	H4	H12	H24	H48	H72	H96
Populationgénérale	1±0,83	1,9±1,06	1,8±0,74	1,5±0,90	0,9±0,58	0,6±0,44	0,5±0,37
population coronaire	0,7±0,32	1,5±0,52	1,7±0,68	1,4±0,94	0,8±0,68	0,5±0,50	0,4±0,39
population valvulaire	1,5±1,13	2,5±1,45	2,1±1,05	1,8±0,80	1±0,31	0,8±0,27	0,6±0,27

**Figure 1:**
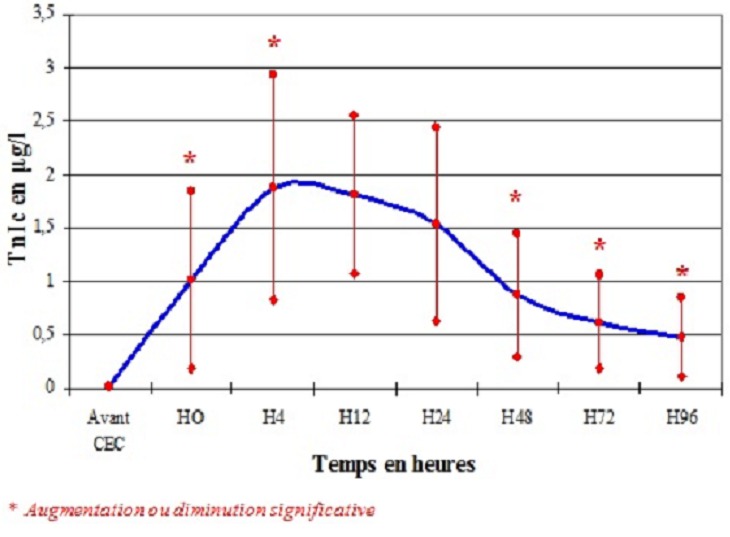
La cinétique de la TnIc de la population générale

**Figure 2:**
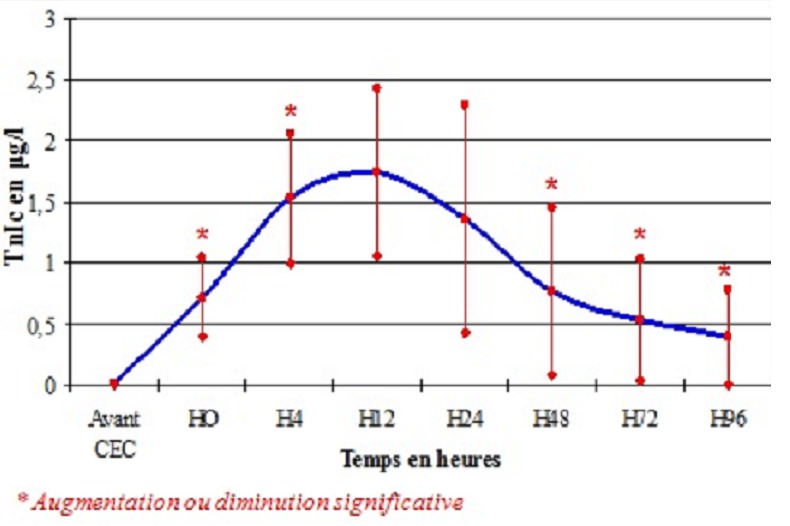
Cinétique de la TnIc post chirurgie coronaire sous circulation extracorporelle

**Figure 3:**
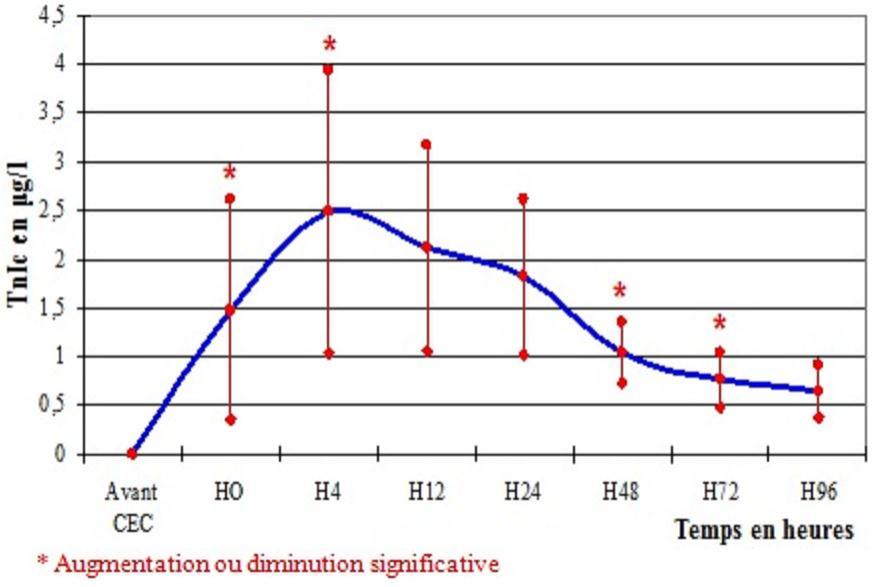
Cinétique de la TnIc en chirurgie valvulaire sous circulation extracorporelle

La cinétique de la TnIc a montré une augmentation significative du taux de la TnIc libéré dès l'arrêt de la CEC jusqu’à un pic entre H4 et H12 après déclampage de l'aorte, puis une diminution progressive (Figure 1). Pour les 21 patients opérés pour pontage coronarien non compliqué d'IDM, les moyennes de la TnIc aux différents temps de dosage sont résumées dans le Tableau 7. Dans cette population, la concentration de la TnIc libérée en post opératoire a augmenté significativement avec un pic à la 12ème heure (valeur maximale: 1,7±0,68 µg/l) puis a diminué significativement dans les 4 jours qui suivaient l'intervention (Figure 2). Les moyennes des taux de la TnIc post chirurgie valvulaire aux différents temps de prélèvement sont résumées dans le Tableau 6. Le même aspect de courbe a été obtenu pour les 13 patients opérés pour chirurgie valvulaire. Cependant, le pic des taux de la TnIc a été retrouvé à H4 (2,5±1,45 µg/l) puis a diminué progressivement (Figure 3).

**Tableau 7:** Cutoff aux différents temps étudiés: population coronaire et valvulaire

Heure	HO	H4	H12	H24	H48	H72	H96
Cutoff coronaire	1,36	2,58	3,10	3,23	2,13	1,53	1,17
Cutoff							
valvulaire	3,75	5,39	4,22	3,41	1,65	1,30	1,19

**Figure 4:**
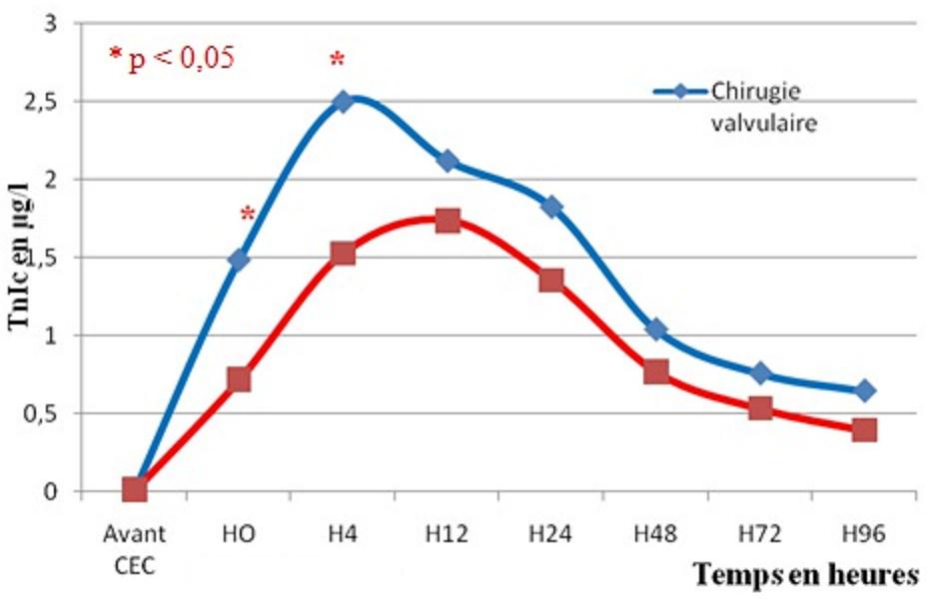
Différence de la libération de TnIc liée aux procédures chirurgicales

Les taux de TnIc obtenus étaient significativement supérieurs dans le cas de la chirurgie valvulaire à H0 et H4 par rapport à la chirurgie coronaire. Pour les 4 premières heures après le déclampage de l'aorte, la quantité de TnIc libérée par heure a été calculée “(valeur H4 - valeur H0)/4”. La TnIc a été 2 fois plus élevée pour une chirurgie valvulaire que pour une chirurgie coronaire (chirurgie valvulaire: 0,4±0,14 µg/l/h; chirurgie coronaire: 0,2±0,10 µg/l/h; p=0,003) et ceci, pour une même durée de clampage (chirurgie coronaire: 60,7±18,3 min; chirurgie valvulaire: 70,8±28,6 min; p=0,209). La comparaison des taux de TnIc aux différents temps de prélèvement est représentée dans la Figure 4.

**Figure 5:**
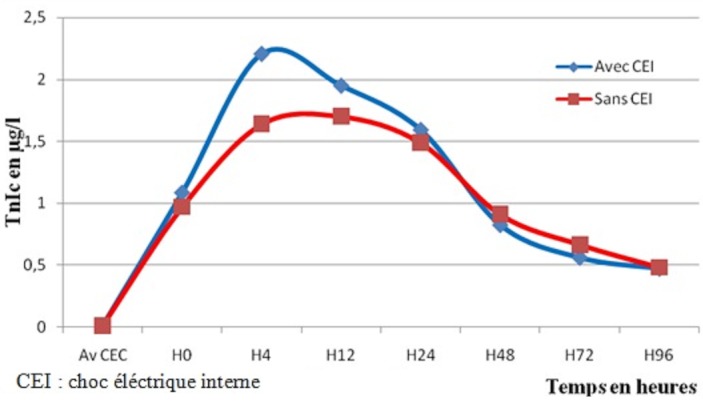
Comparaison du taux de TnIc lié a l'utilisation ou non de choc électrique externe chez la population générale

Quinze patients (44,1%) de la population générale ont reçu un ou plusieurs chocs électriques internes à la fin de la CEC. L'analyse de la cinétique des moyennes de TnIc chez ces patients, aux différents temps, a montré une augmentation non significative à H4 jusqu'au H12 (Figure 5). Les taux de TnIc à H4 ont été positivement corrélés au nombre de chocs électriques internes reçus (r=0,370; p=0,037).

**Figure 6:**
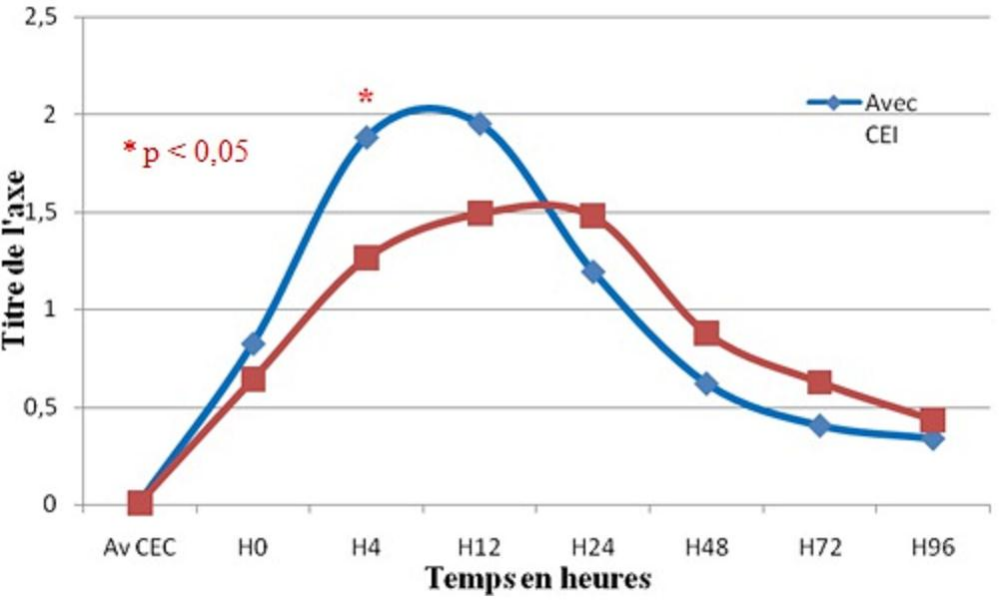
Comparaison du taux de TnIc lié a l'utilisation ou non de choc électrique externe chez la population coronaire

La comparaison, selon le type de chirurgie, des moyennes de TnIc chez les patients ayant reçu un ou plusieurs CEI et ceux n'en ayant pas reçu n'a pas montré pas de différence en chirurgie valvulaire. Cependant, pour la population coronaire, il a été constaté une augmentation significative de la concentration moyenne de TnIc à H4 chez les patients ayant reçu des CEI (1,9±0,6 µg/l contre 1,3±0,3µg/l, p=0,004) (Figure 6).

Le Tableau 7 montre les cutoff chez la population coronaire et la population valvulaire aux différents temps étudiés. Tous les patients indemnes de complications cardiaques avaient des valeurs inférieures au “cutoff” aux différents temps de prélèvements étudiés.

## Discussion

Les protéines spécifiques des cellules musculaires cardiaques telles que les troponines ont fait l'objet de nombreuses études et ils sont largement utilisés comme marqueurs dans le diagnostic et le pronostic des pathologies cardiaques [1]. Parmi ces protéines, les troponines présentent l'avantage d'avoir des isoformes cardiospécifiques, ce qui permet leur utilisation dans le diagnostic des pathologies cardiaques, même en présence d'atteinte organique d'origine musculaire.

Dans notre étude on a suivi la cinétique des troponines Ic, un isoforme cardiospécifique, après une chirurgie cardiaque sous CEC non compliqué d'infarctus du myocarde post opératoire. On a trouvé que les valeurs de TnIc augmentent en postopératoire immédiat (dès l'arrêt de la CEC), pour atteindre un maximum à H4 puis diminuent progressivement pour se normaliser dans les 4 à 5 jours qui suivent l'intervention et ceci en dehors de complications cardiaques post opératoire. Nos résultats sont comparables avec ceux d'autres études [3, 4] qui ont étudiés la cinétique des troponines suite à une chirurgie cardiaque avec clampage aortique. Ces études ont montrés que la TnIc augmente et atteint un pic 6 à 12 heures après le déclampage aortique. Le retour à la normale, survient 5 à 7 jours après la chirurgie.

En chirurgie cardiaque comme la chirurgie valvulaire ou le pontage aorto-coronaire, une atteinte mécanique est constante, suite aux manipulations directes du muscle, aux décharges et canulations et peut-être minorée par la technique de cardioplégie. Les sensibilités et spécificités de la TnIc expliquent l'observation de valeurs augmentées dans la période postopératoire, même en l'absence de complications [5, 6]. Dans notre étude, nous avons comparé les moyennes de TnIc libérée en postopératoire selon le type de chirurgie. Les taux de TnIc augmentent significativement dés le déclampage aortique avec un pic à H4 pour la chirurgie valvulaire (2,5±1,13 µg/l) et un pic à H12 pour la chirurgie coronaire (1,7±0,68 µg/l) toujours en dehors de contexte d'IDM périopératoire. La quantité de TnIc libéré par heure a été 2 fois plus élevée pour une chirurgie valvulaire (0,4±0,14 µg/l/h) que pour une chirurgie coronaire (0,2±0,10 µg/l/h) et ceci pour une même durée de clampage aortique. Nos résultats rejoignaient ceux de Cauliez et collaborateurs [7] qui ont trouvé que les concentrations de TnIc sont maximales à la 24ème heure en cas de pontage coronarien (valeur maximale: 3,3±0,7 µg/l) et de remplacement valvulaire (5,0±0,6 µg/l). De plus, la quantité de TnIc libérée dans les 3 heures suivant l'intervention est 2,5 fois plus importante après remplacement valvulaire et ceci, pour une même durée de clampage. Ceci est lié aux lésions myocardiques directement induites par la procédure chirurgicale. En revanche, Vermes et collaborateurs [8] ont montré que les pics de TnIc postopératoires étaient à la 12ème heure post déclampage aortique sans aucune différence significative entre les moyennes de la TnIc des deux groupes.

Il faut noter que l'interprétation du taux de troponine est en fonction du contexte clinique et du type d'intervention. Bien évidemment, lorsqu′on s'intéresse à des chirurgies plus complexes (avec CEC ou clampage aortique) et nécessitant un traumatisme musculaire plus important, l′élévation de troponine est plus importante [9]. Aussi, en plus du traumatisme chirurgical la CEC joue un rôle majeur dans l’élévation post opératoire des troponines. La circulation extracorporelle implique une souffrance myocardique. Jai S. Raman et collaborateurs [10], ont trouvé une corrélation significative entre les taux de la TnIc libérée et les durées de clampage aortique et de la CEC. De même, Cauliez et collaborateurs [7], ont trouvé que les concentrations de TnIc sont positivement corrélées à la durée du clampage aortique. Cependant, cette corrélation n'est pas significative dans notre étude. Ce ci peut être expliqué par la petite taille de notre échantillon.

Dans notre étude, nos résultats ont montré que les taux de TnIc à H4 étaient positivement corrélés au nombre de chocs électriques internes reçus (r=0,370; p=0,037). Ces résultats étaient en accord avec ceux de Rodriguez-Castro et collaborateurs [11] qui ont trouvé que les taux de la TnIc présentaient une augmentation significative supplémentaire chez les patients ayant reçu plus de deux chocs électriques internes post CEC.

Pour trancher entre infarctus et dommage myocardique, des valeurs seuils dites “cutoff” spécifiques au type de la chirurgie et propres à chaque équipe doivent être recherchés [12, 13]. Ces cutoff sont spécifique à chaque équipe car la technique de dosage de la TnIc est encore non standardisée et il existe de grandes variations entre les différentes méthodes de dosage. En effet, on peut constater jusqu’à un facteur 20 de variation pour le taux de TnIc d'un même prélèvement en fonction des trousses de dosage utilisées [14] Des différences de résultats ont été mises en évidence selon l'anticoagulant utilisé pour le prélèvement [15]. Dans notre étude, Tous les prélèvements ont été effectués dans des tubes à l'héparinate de lithium. L'utilisation de plasma hépariné est recommandée en raison de la grande rapidité d'exécution et du risque de résultats faux positifs par présence de particules de fibrine. [16]. La centrifugation insuffisante [17], le défaut de conservation du prélèvement ou la coagulation incomplète [18] du sérum peuvent aboutir à des faux positifs. De même, la présence d'anticorps hétérophiles peut entraîner une fausse positivité de la TnIc [19].

Il existe plusieurs méthodes de dosages de la TnIc dans le sérum basées sur la sélection d'anticorps reconnaissant des épitopes spécifiques. Ces immunodosages entièrement automatisés diffèrent à la fois par la sélection des couples d'anticorps utilisés, par la spécificité différente de ces anticorps, et par des conditions variées de réaction ce qui explique l'hétérogénéité des seuils décisionnels. Cette hétérogénéité peut être expliquée en partie par l'absence de correspondance entre les systèmes antigène-anticorps utilisés par les différents fabricants. Malgré qu'il existe une corrélation satisfaisante entre les différents immunodosages [15], l'absence actuelle de standardisation des dosages de la TnIc rende difficile, voire impossible, la comparaison des résultats.

Il est évident qu′en cas d′infarctus péri opératoire les taux de troponine observés sont élevés. En cas d′IDM “significatif” avec des ondes Q de nécrose à l′ECG, le pic de TnIc apparaît 20 à 24 heures après la fin de la chirurgie. En l′absence d′IDM documenté, la TnIc augmente plus tôt et les concentrations observées sont plus basses [20].

Il faut donc dans la chirurgie cardiaque, établir des “cutoff”, propres à chaque équipe, bien que les premiers résultats publiés soient tous assez concordants: un taux entre 10 et 15 µg/l semblent être la valeur frontière entre patients indemnes et patients atteints de complications cardiaques en chirurgie coronaire [21]. Onorati et collaborateurs [12], ont défini la présence de lésions myocardiques lorsque le taux de TnIc à T12 après le déclampage aortique dépasse le seuil de 3,1 µg/l. Cependant, Mair et collaborateurs [22], ont conclu dans leur étude qu'un pic sérique de TnIc à T6 supérieur à 3,7 µg/l et des concentrations de TnIc supérieures à 3,1 µg/l à T12 et à 2,5 µg/l à T24 après le déclampage aortique traduisent très probablement un IDM péri opératoire. Dans l’étude de Cauliez et collaborateurs [7], chez les patients ayant présenté un infarctus postopératoire, la concentration maximale est obtenue à la 24ème heure après l'intervention pour la troponine Ic. Le diagnostic d'une telle pathologie peut être établi avec une sensibilité de 100% à la 24ème heure avec une valeur seuil de 7 µg/L pour la TnIc sur les analyseurs Stratus (Dade-Behring).

Dans notre étude, nous avons calculé un cutoff propre à notre équipe et à notre laboratoire pour chaque type de chirurgie aux différents temps de prélèvement, ce qui nous a permis de faire la différence entre le diagnostic d'IDM péri opératoire et celui du dommage myocardique lié aux procédures chirurgicales. Le “cutoff” a été défini, pour chaque population, comme la valeur moyenne plus deux déviations standards des patients indemnes de complications cardiaques. Le diagnostic d'Infarctus per ou postopératoire immédiat peut être retenu en cas d’élévation de la TnIc au-dessus de la valeur seuil “cutoff”. Alors qu'en cas d’élévation intermédiaire du marqueur qui reste toujours en dessous du seuil diagnostique d'infarctus, on parle alors de dommage myocardique lié à la procédure chirurgicale.

## Conclusion

L'IDM péri opératoire constitue une complication redoutable lors de chirurgie cardiaque. La TnIc, isoforme spécifique du c'ur de troponine I [1], perd sa spécificité pour ce diagnostic vue son augmentation secondaire au dommage myocardique même en dehors de tout contexte d'infarction. Le diagnostic d'IDM péri opératoire devrait tenir compte de cette cinétique. La connaissance de la cinétique de TnIc lors de chirurgie cardiaque non compliquée et la fixation de valeur seuil ou "Cutoff "permet aux cliniciens de distinguer entre dommage myocardique secondaire à la chirurgie et IDM [1, 6]. Notre étude a permis d’étudier la cinétique normale de la TnIc et d’établir ces valeurs seuils spécifique pour notre établissement, afin d'envisager précocement la stratégie thérapeutique la plus adéquate pour nos malades
